# Intraperitoneal Triamcinolone Reduces Postoperative Adhesions, Possibly through Alteration of Mitochondrial Function

**DOI:** 10.3390/jcm11020301

**Published:** 2022-01-07

**Authors:** Neeraja Purandare, Katherine J. Kramer, Paige Minchella, Sarah Ottum, Christopher Walker, Jessica Rausch, Conrad R. Chao, Lawrence I. Grossman, Siddhesh Aras, Maurice-Andre Recanati

**Affiliations:** 1 Center for Molecular Medicine and Genetics, School of Medicine, Wayne State University, Detroit, MI 48201, USA; neeraja.purandare@wayne.edu (N.P.); lgrossman@wayne.edu (L.I.G.); saras@wayne.edu (S.A.); 2 Department of Obstetrics and Gynecology, St. Vincent’s Medical Centers Manhattan, New York, NY 10011, USA; katherinekramer@gmail.com; 3 Department of Molecular and Integrative Physiology, Kansas University Medical Center, Kansas City, KS 66160, USA; pminchella@kumc.edu; 4 Department of Obstetrics and Gynecology, University of Cincinnati, Cincinnati, OH 45267, USA; ottumse@ucmail.uc.edu; 5 Department of Gynecologic Oncology, University of Texas Southwestern Medical Center, Dallas, TX 75390, USA; cwalker0812@gmail.com; 6 Department of Obstetrics and Gynecology, Hutzel Hospital, Detroit Medical Center, Detroit, MI 48201, USA; jrausch@med.wayne.edu; 7 Department of Obstetrics and Gynecology, University of New Mexico, Albuquerque, NM 87131, USA; verdi@earthlink.net; 8 Department of Obstetrics and Gynecology, Wayne State University, Detroit, MI 48201, USA

**Keywords:** myomectomy, adhesions, fibroblast, mitochondria, TGF-β1, ROS

## Abstract

Adhesions frequently occur postoperatively, causing morbidity. In this noninterventional observational cohort study, we enrolled patients who presented for repeat abdominal surgery, after a history of previous abdominal myomectomy, from March 1998 to June 20210 at St. Vincent’s Catholic Medical Centers. The primary outcome of this pilot study was to compare adhesion rates, extent, and severity in patients who were treated with intraperitoneal triamcinolone acetonide during the initial abdominal myomectomy (*n* = 31) with those who did not receive any antiadhesion interventions (*n* = 21), as documented on retrospective chart review. Adhesions were blindly scored using a standard scoring system. About 32% of patients were found to have adhesions in the triamcinolone group compared to 71% in the untreated group (*p* < 0.01). Compared to controls, adhesions were significantly less in number (0.71 vs. 2.09, *p* < 0.005), severity (0.54 vs. 1.38, *p* < 0.004), and extent (0.45 vs. 1.28, *p* < 0.003). To understand the molecular mechanisms, human fibroblasts were incubated in hypoxic conditions and treated with triamcinolone or vehicle. In vitro studies showed that triamcinolone directly prevents the surge of reactive oxygen species triggered by 2% hypoxia and prevents the increase in TGF-β1 that leads to the irreversible conversion of fibroblasts to an adhesion phenotype. Triamcinolone prevents the increase in reactive oxygen species through alterations in mitochondrial function that are HIF-1α-independent. Controlling mitochondrial function may thus allow for adhesion-free surgery and reduced postoperative complications.

## 1. Introduction

The development of adhesions is a commonly documented finding in patients with a history of abdominal surgery. These fibrous bands can lead to bowel obstruction, chronic pain, dyspareunia, infertility, fistulas, and complications at the time of reoperation [1,2,3]. Yearly, there are over 300,000 adhesion-related hospitalizations, costing $2.3 billion [4,5]. Nearly half involve the reproductive tract of women [4,6,7]. Adhesions occur regardless of surgical route [8,9,10] and frequently occur after Cesarian sections [11,12] or gynecological surgery [13].

Among gynecological surgeries, myomectomy is an important cause of abdominal adhesions [14,15]. Leiomyomas are common benign tumors with a prevalence of 20–40% in reproductive-age women [16,17] and 70–80% in women over age 50, with higher rates in African-Americans [18]. A quarter of women with fibroids become symptomatic [19] and experience infertility, urinary symptoms, pelvic pressure, pain, or abnormal uterine bleeding [20]. Leiomyomas are, therefore, a significant issue in women’s health.

The treatment of symptomatic uterine fibroids depends on the number, location, patient’s overall health, personal desires, and reproductive choices. Approaches include gonadotropin-releasing hormone (GnRH) agonists [21,22], aromatase inhibitors [23], selective estrogen receptor modulators [24], oral contraceptives, progesterone intrauterine devices [25], and anti-inflammatory drugs [26], as well as uterine artery embolization [27], MRI-focused ultrasound [28], and radio frequency ablation [29]. Despite the availability of these more conservative approaches, the standard approach to the treatment of symptomatic fibroids remains surgical. Leiomyomas are the leading indication for hysterectomy [30,31]. However, for women desiring to preserve their uterus or maintain fertility, myomectomy remains the most practical option [32,33,34]. Rates of operations range from 1.3 to 9.2/10,000 per year in white and African-American women, respectively [35]. Although a minimally invasive surgical approach is preferred [36], the number, size, and location, as well as the experience and preference of the surgeon, dictate how the procedure will be performed [22]. In 2014, FDA regulations regarding morcellation influenced surgical approaches and 60% of large myomectomies are now performed through open procedures [37].

As abdominal surgery, particularly myomectomy, causes high rates of adhesions and because this surgery is routinely performed, we set out to study the reduction in adhesion formation after myomectomy using one particular surgical technique [38,39]. In this noninterventional observational cohort study, we compare adhesion rates in patients who were treated with intraoperative, heparinized saline irrigation with intraperitoneal triamcinolone acetonide and dextran during a previous myomectomy with patients who did not receive any anti-adhesion interventions whatsoever. This steroid has been routinely used during abdominal myomectomies in women with very large uteri [39] due to the anti-inflammatory properties of triamcinolone, it being relatively inexpensive and readily available, and the anecdotal results obtained from gynecologists who have used it clinically. Patients who presented for repeat surgery, years later, consented to the observational study, and the adhesions were evaluated at that time. The hypothesis was that steroid-treated patients would exhibit less fibrosis, and the primary outcome, the number of patients with postoperative adhesions, would be reduced as well as the overall burden of adhesion. Subsequently, and more importantly, using an in vitro model, we sought to determine the molecular mechanisms through which steroids may decrease the conversion of fibroblasts to the adhesion phenotype, thereby decreasing adhesion formation.

## 2. Materials and Methods

### 2.1. Patient Selection

**Figure 1:**
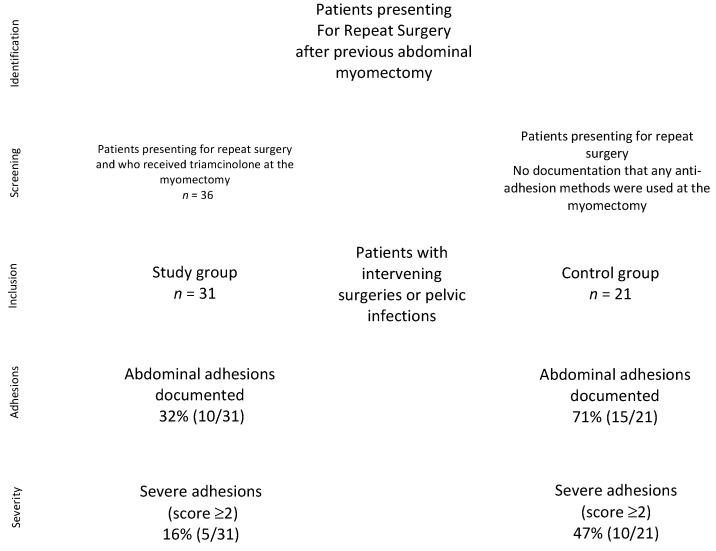
Study design and patient inclusion.

Under IRB approval #010419M1X, admissions for repeat abdominal surgery, after a history of previous abdominal myomectomy, from 1 March 1998 to June 2010 at St. Vincent’s Catholic Medical Center, Manhattan, were asked to participate in this noninterventional observational retrospective study. Signed written informed consent was obtained on the day of surgery. This study did not meet the NIH definition of a clinical trial. Thirty-six cases were reoperations of patients who had received 200 mg intraperitoneal triamcinolone acetonide in 500 mL dextran and no adhesion barriers (such as Seprafilm) at the time of the original abdominal myomectomy, as previously published [39]. In this group, five patients were removed for intervening Cesarian delivery or for presumed pelvic inflammatory disease (PID), leaving *n* = 31 study patients. For the control group, we excluded patients who had received any form of anti-adhesion measures (such as barriers, heparin), as documented in the operative note, intervening surgeries, or abdominal infections, leaving *n* = 21 patients. Patients who had prior adhesions or endometriosis documented on the initial surgery, and those who had abdominal surgery or PID since the initial surgery, were excluded from both arms of the study (Figure 1). Patients were evaluated preoperatively with pelvic ultrasound, endometrial biopsy, cervical cytology, blood counts, thyroid function, and a pregnancy test. Data from the original myomectomy for consented patients were obtained from the hospital records office and from office charts.

### 2.2. Surgical Evaluation of Adhesions

Upon entry, the peritoneum was examined for adhesions, and their location, number, and severity were recorded. Adhesions were scored by the assistant, with the concurrence of the surgeon, who was blinded as to the patient having previously received triamcinolone at the original surgery or the length of time elapsed from this surgery. The assistant was previously trained in using the scoring system described in Table 1.

### 2.3. Cell Culture and Hypoxia Experiments

Experiments were performed with human female fibroblasts (a kind gift from Prof. Michael Tainsky, Wayne State University) cultured in Dulbecco’s modified Eagle’s medium (DMEM) (HyClone) with 10% fetal bovine serum (FBS) (Sigma Aldrich, St. Louis, MO, USA) and 1% penicillin–streptomycin (HyClone). To achieve hypoxic conditions, the incubation chamber was placed at 37 °C and infused with CO_2_ and N_2_. Gas flow was controlled with a proOX110 gas controller (BioSpherix, Redfield, NY, USA) to achieve 1% or 2 % oxygen and 5% CO_2_. Oxygen equilibration time (3–4 h) was accounted for in the hypoxia experiments.

### 2.4. Measurement of Reactive Oxygen Species

Total cellular ROS measurements were performed with CM-H_2_DCFDA (Life Technologies, Grand Island, NY, USA) or ROS-Glo (Promega, Madison, WI, USA). For CM-H_2_DCFDA measurements, cells were split into 12-well plates at 2 × 10^5^ cells per well and incubated for 24 h with vehicle or indicated concentrations of triamcinolone acetonide. Cells were then treated with CM-H_2_DCFDA at a final concentration of 10 μM, washed twice in phosphate-buffered saline, and analyzed for fluorescence (Ex: 485 nm/Em: 527 nm) on a Gen5 microplate reader (BioTek Inc, Winooski, VT, USA). For ROS-Glo measurements, cells were distributed into 96-weRll plates at 2 × 10^4^ cells per well and incubated for 24 h with vehicle or 1 mM triamcinolone acetonide (Teva Pharmaceuticals). Cells were co-treated with 2 μM paraquat (SCBT, Dallas, TX, USA) and the H_2_O_2_ substrate for 2 h, followed by the addition of the detection agent and the measurement of luminescence according to the manufacturer’s instructions.

### 2.5. Measurement of Intact Cellular Oxygen Consumption

Intact cellular oxygen consumption was measured with a Seahorse XF^e^24 bioanalyzer (Agilent, Santa Clara, CA, USA). Cells were plated at a concentration of 2 × 10^4^ per well and incubated for 24 h with vehicle or 1 mM triamcinolone acetonide. Paraquat (2 µM) was added for 2 h, and basal oxygen consumption measurements were performed according to the manufacturer’s instructions.

### 2.6. ELISA

Supernatants from human fibroblasts treated with triamcinolone or vehicle were used for the measurement of human TGF-β1 levels using an ELISA kit (Boster Biological Technology, Pleasanton, CA, USA) per the manufacturer’s instructions.

### 2.7. Immunoblotting

Immunoblotting on a PVDF membrane was performed as previously described [40,41]. Unless specified otherwise, primary antibodies were used at a concentration of 1:500 and secondary antibodies at a concentration of 1:5000. We obtained anti-HIF1α and anti-SOD2 antibodies (Proteintech, Chicago, IL, USA), anti-NOX2 (GeneTex, Irvine, CA, USA), and anti-Actin and anti-Tubulin antibodies (Cell Signaling Technology, Danvers, MA, USA) from the indicated sources.

### 2.8. Statistical Analysis

For clinical analysis, deidentified data were collected in an Excel spreadsheet and analyzed using Statistical Package for the Social Sciences (SPSS) (Version 27, IBM Analytics, Armonk, NY, USA). Descriptive statistics were used to characterize the sample. The Kolmogorov-Smirnov normality test was applied to all variables. Welch’s t-test was performed on normal data to compare groups (independent samples, two-tailed, and both equal and non-equal variance), while non-normal groups were compared using the Mann-Whitney U-test. The Pearson chi-squared test was performed on categorical data. A value of *p* < 0.05 was considered significant. Separately, a Pearson correlation was performed, with *p*-value analysis, and r from −1.0 to −0.5 or from 1.0 to 0.5 was considered correlated. The sample size needed for a 40% reduction in the rate of adhesions was *n* = 58 total patients based on an 88% background rate of adhesions, 80% power, and α = 0.05 (sample-size.net).

For in vitro experiments, statistical analyses were performed with MSTAT version 6.1.1 (N. Drinkwater, University of Wisconsin, Madison, WI, USA). The two-sided Wilcoxon rank-sum test was applied to determine statistical significance for *p*-values. Data were considered statistically significant with *p* < 0.05. The *n* number corresponds to biological replicates, and statistical analysis was performed where *n* ≥ 3.

### 2.9. Ethics Approval

This retrospective noninterventional observational cohort study was approved by the institutional review board at St. Vincent’s Catholic Medical Centers, Manhattan, from its inception in February 1998 until June 2010, when the institution closed, and subsequently by Wayne State University since 3 January 2019 (IRB #010419M1X). All procedures performed in studies involving human participants were in accordance with the ethical standards of the institutional and national research committee and with the 1964 Declaration of Helsinki and its later amendments or comparable ethical standards.

## 3. Results

### 3.1. Sample Population

There were no statistical differences between the control and study groups with respect to age, uterine size at reoperation, number and aggregate weight of fibroids removed, operative time, blood loss, and mean time to reoperation (Table 2). Indications for reoperation in the study group principally included abdominal/pelvic pain, menorrhagia, and infertility, with many patients having more than one indication.

### 3.2. Effect of Triamcinolone Intervention on Adhesions and Surgical Complications

In the study group, 32% of patients (*n* = 10) were found to have adhesions compared to 71% (*n* = 15) in the control group, a significant difference (*p* < 0.01). When compared to the controls, adhesions in the study group were significantly fewer in number (0.71 vs. 2.09, *p* < 0.005), severity (0.54 vs. 1.38, *p* < 0.004), and extent (0.45 vs. 1.28, *p* < 0.003).

Lysis of adhesions was performed successfully in all patients. Surgical complications in the study group included one patient (3%) who experienced a cystostomy as a result of adhesions, whereas three patients (14%) in the control group experienced surgical complications due to severe adhesions near the bladder dome (*n* = 2) or the bowel (*n* = 1). In all cases, these injuries were repaired intraoperatively (Table 2).

### 3.3. Risk Factors for Adhesion Formation and Severity of Adhesions in the Study Group

Stratifying the study group, adhesions were absent when, at original myomectomy, the uterus was <22-week size (*p* < 0.001), EBL was <500 mL (*p* < 0.001), or surgical time was <200 min (*p* < 0.001) (Table 3). Conversely, the presence of posterior (*p* < 0.001) or cervical (*p* < 0.001) fibroids was significantly correlated with the presence of adhesions (not shown).

Adhesion severity ≥ 2 (i.e., worse than filmy adhesions) was significantly correlated with EBL, surgical time, number of fibroids removed (*p* < 0.05) (Figure S1) as well as posterior or cervical location and uterine size (*p* < 0.05) (Table S1). Race was not related to the presence of adhesions, adhesion numbers, severity, or extent of adhesions. The average time interval between surgeries of 7.7 years (range from 5–15) did not correlate with the number, extent, severity, or location of adhesions.

### 3.4. In Vitro Cell Culture Model of the Role of Steroids in Fibrosis

#### 3.4.1. HIF-1α Does Not Affect TGF-β1 Secretion in Human Fibroblasts at 1% Hypoxia or after Reoxygenation

**Figure 2:**
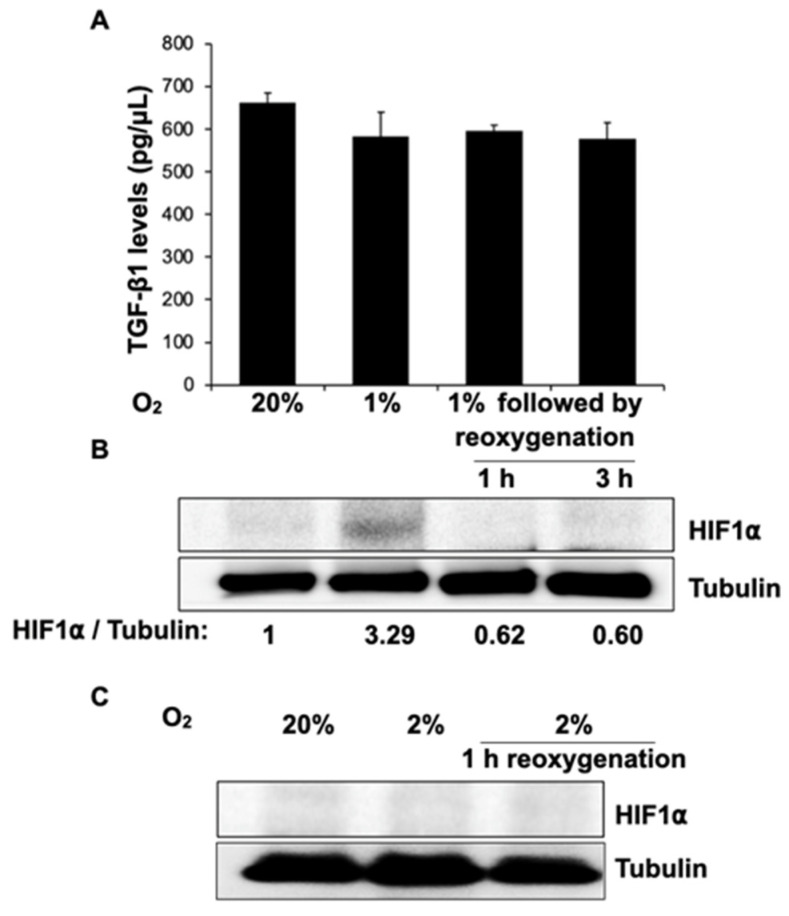
HIF-1a does not affect TGF-b1 secretion in human fibroblasts at 1% or 2% hypoxia or reoxygenation. Human fibroblasts were incubated at 20% O_2_, 1 % O_2_, or 1 % O_2_ for 24 h, followed by reoxygenation for 1 or 3 h. (**A**) Equal amounts of cell culture supernatants from these were used to measure TGF-β1 levels using ELISA. Error bars on the graph indicate standard deviation from the mean (*n* = 2). (**B**) Equal amounts of cell lysates from the experiment in (**A**) were separated on an SDS-PAGE gel and probed for HIF1α levels. Tubulin was probed as loading control (immunoblot below). While HIF1α increased with hypoxia and decreased following reoxygenation, no change was observed in TGF-β1 levels. (**C**) Similarly, fibroblasts were incubated at 20% O_2_, 2 % O_2_, or 2 % O_2_ for 24 h, followed by reoxygenation for 1 h, and probed for HIF1α levels. HIF1α was not present at 2% hypoxia.

Observing a decrease in adhesion formation in vivo, we set out to determine the cellular mechanisms by which steroids modulate fibrosis using a fibroblast cell culture model. TGF-β1 is the most potent profibrogenic cytokine [42], and it irreversibly converts fibroblasts to an adhesion phenotype [43]. Based on animal models, many groups have suggested that tissue hypoxia of less than ~7 mm Hg (or ~1% hypoxia) [2], found during minimally invasive surgery [44] and postoperatively in ischemic tissues [45], triggers fibrosis through a HIF-1α pathway [2,45,46,47]. To test this hypothesis, we incubated fibroblasts at 20% or 1% oxygen for 24 h and analyzed TGF-β1 levels from cell culture supernatants using an enzyme-linked immunosorbent assay (ELISA). We similarly measured TGF-β1 following 1 h and 3 h reoxygenation (20% oxygen) of cells that had undergone 24 h hypoxia at 1% oxygen to test if hypoxia–reoxygenation triggers TGF-β1 secretion (Figure 2A). To confirm that 1% oxygen levels were achieved in the hypoxia chamber, cell lysates from the same experiment were assessed for HIF-1α levels (Figure 2B) using Western blot. While cells incubated at 1% O_2_ showed a ~three-fold increase in HIF-1α compared to cells at 20% O_2_, no change in TGF-β1 levels was seen. Reoxygenation also did not alter TGF-β1 levels. Thus, HIF-1α is unlikely to affect TGF-β1 secretion in fibroblasts.

Although no data are available in humans, normal mammalian peritoneal oxygen tension [48,49] is about 8% [50]. As no differences in the expression of adhesion phenotype markers have been found in the range of 8–20% [50], controls were cultured in 20% O_2_. Because the peritoneal levels of O_2_ that result from surgical tissue injury are not known, data taken from animal models that suggest abraded tissues as well as cut peritoneum exhibited O_2_ levels of about 2% [51]. Based on this, we checked for the presence of HIF-1α at 2% hypoxia and found that it was not present (Figure 2C), suggesting a HIF-independent pathway.

#### 3.4.2. Triamcinolone Prevents the Increase in TGF-β1 Resulting from 2% Hypoxia

**Figure 3:**
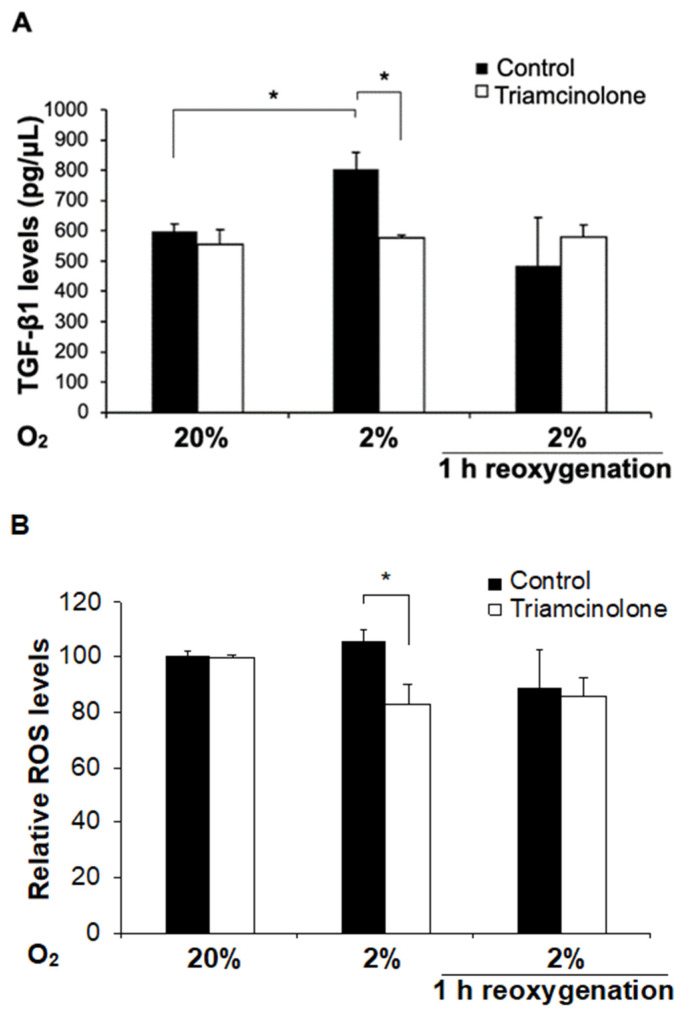
At 2% hypoxia, triamcinolone decreases reactive oxygen species formation and prevents TGF-b1 secretion while not having an effect at normoxia or after reoxygenation. Human fibroblasts treated with either vehicle or 1 mM triamcinolone were incubated at 20% or 2% O_2_ for 24 h or 2% O_2_, followed by reoxygenation for 1 h. (**A**) Equal amounts of cell culture supernatants were used to measure TGF-β1 levels using ELISA (*n* = 4 for 20% and 2%, *n* = 2 for 2% reoxygenation). * denotes *p* < 0.05, ns for rest. (**B**) Total cellular ROS (reactive oxygen species) levels were measured using CM-H_2_DCFDA (*n* = 4). * denotes *p* = 0.028 for 2% control vs. triamcinolone only; ns for rest.

Subsequently, we examined the effect of triamcinolone on fibroblasts at 2% hypoxia as well as controls at 20% oxygen. Fibroblasts were treated with either 1 mM triamcinolone or vehicle for 24 h, and equal amounts of cell culture supernatants from these were used to measure TGF-β1 levels. This concentration was similar to that administered intraperitoneally to patients, as described earlier in our noninterventional observational cohort study [39]. We found that 2% hypoxia alone significantly increased TGF-β1 levels relative to controls, but in cells treated with triamcinolone, the TGF-β1 levels did not increase. We then re-exposed the cells that had been hypoxic (2% oxygen for 24 h) back to 20% oxygen for 1 h and did not detect a change in TGF-β1 levels relative to controls (Figure 3A).

#### 3.4.3. Triamcinolone Decreases Reactive Oxygen Species (ROS) Only at 2% Hypoxia

There has been increased recognition that the pathogenesis of adhesion development includes contributions of hypoxia and/or hyperoxia at the site of surgery, resulting in oxidative stress and the production of ROS [52,53,54]. ROS can be generated by cytoplasmic ROS-generating enzymes such as NADPH oxidase (NOX) or mitochondrial electron transport chain complexes I or III [55,56,57,58]. Deficits that affect mitochondrial ROS generation, such as mitochondrial DNA mutations, have been implicated in supporting the growths of cancers [59], which may also be common to the formation of adhesions. High ROS levels also may activate plasma membrane proteins (such as TRPC6 [60]) that are important in ischemic stroke [61], a condition where hypoxia plays a significant role. To investigate a link between hypoxia and ROS, we asked if human fibroblasts exposed to 2% hypoxia alter ROS levels. We measured these using CM-H_2_DCFDA and found that similar to TGF-β1, 2% hypoxia incubation for 24 h generated minimal ROS but triamcinolone-treated cells had significantly reduced basal ROS levels relative to controls. Returning the cells exposed to 2% hypoxia to 20% oxygen did not vary ROS levels significantly. Furthermore, triamcinolone (at 1 mM) had no effect on ROS at 20% oxygen levels whether they remained at 20% (controls) or underwent hypoxia or hypoxia followed by reoxygenation (Figure 3B).

#### 3.4.4. Triamcinolone Prevents the Increase in ROS through Alteration of Mitochondrial Function

**Figure 4:**
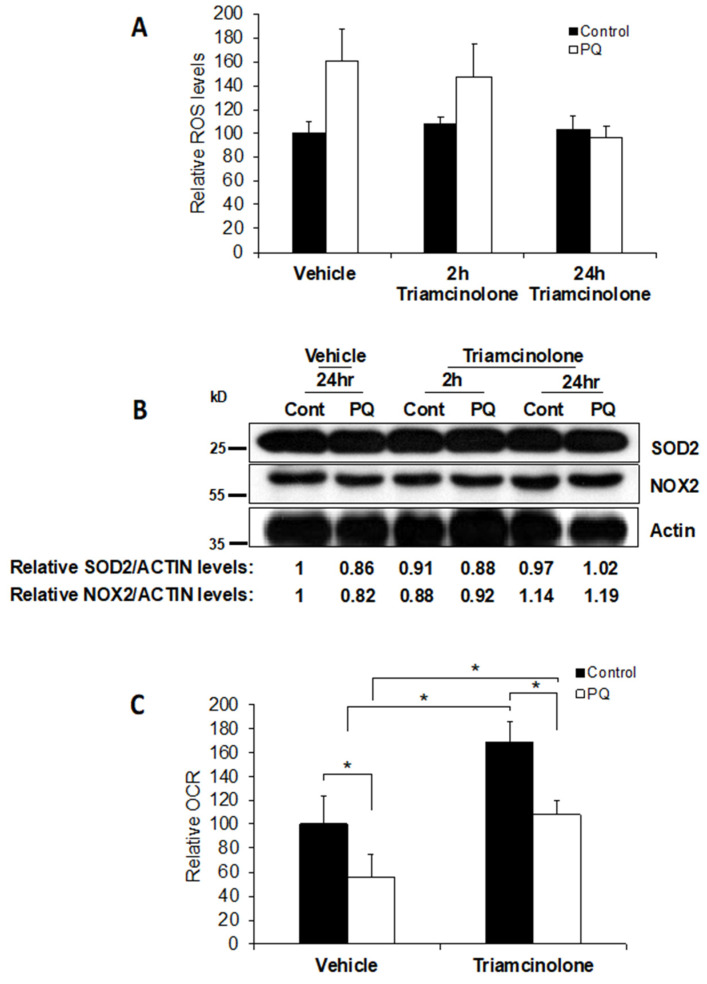
Triamcinolone prevents the increase in ROS through alterations in mitochondrial function. (**A**) Human fibroblasts (1 × 10^5^ cells/well in a 12 well plate) were incubated with vehicle or 1 mM triamcinolone for 24 h and were treated with 2 µM paraquat for 2 h. Cells in the middle group were co-treated with PQ (Paraquat) and triamcinolone for 2 h. ROS levels were measured using the ROS-Glo assay. Error bars on the graph indicate standard deviation from the mean (*n* = 2). (**B**) Equal amounts of cell lysates (from pooled samples in A) were separated on an SDS-PAGE gel and probed for SOD2 and NOX2 levels. Actin was probed as loading control. (**C**) Human fibroblasts (2 × 10^4^ cells/well) were incubated with vehicle or 1 mM triamcinolone for 24 h and then were treated with 2 µM paraquat for 2 h. OCR was measured using the Seahorse bioanalyzer. Error bars on the graph indicate standard deviation from mean (*n* = 4, * indicates *p* < 0.05).

We then asked how triamcinolone prevented the increase in ROS triggered by 2% hypoxia. Possibilities included: direct action of the steroid in capturing ROS, upregulation of ROS scavengers [43], reduction of cytoplasmic ROS generated by NOX2, or reduction of ROS by alteration of mitochondrial function. To test the first two hypotheses, we incubated fibroblasts at 20% oxygen with either vehicle or 1 mM triamcinolone for 24 h prior to treating them with 2 µM paraquat, a ROS inducer, for 2 h. Using the ROS-Glo assay, we established that paraquat significantly increased ROS levels in untreated controls as well as in fibroblasts co-treated with triamcinolone. However, 24 h pretreatment with triamcinolone prevented the increase in ROS caused by paraquat. These results suggest that the steroid did not act as a direct scavenger of ROS but may have modulated certain proteins over the 24 h treatment (Figure 4A).

To determine if triamcinolone had upregulated ROS scavengers, we tested cell lysate SOD2 levels; however, we found no changes in these levels. Next, we asked if the levels of an enzyme that generates ROS in the cytoplasm, NOX2, were changed. We found no significant change in NOX2 either, suggesting that the reduction of ROS observed was not due to increased scavenging activity by SOD2 or cytoplasmic ROS generation by NOX2 (Figure 4B).

Besides cytoplasmic ROS, mitochondria also generate ROS during electron transport chain function. Therefore, we tested whether triamcinolone prevents increased ROS generation by altering mitochondrial function. Using paraquat as a rapid inducer of ROS, we analyzed the effect of triamcinolone. We found that triamcinolone increases the oxygen consumption rate (OCR) whether or not paraquat is added (Figure 4C, bar 1 vs. bar 3). Paraquat addition, however, decreases OCR in each case (Figure 4C, bars 2 and 4). These results suggest that triamcinolone enhances mitochondrial OCR and hence reduces the ROS generated.

## 4. Discussion

### 4.1. Adhesions

In this study, we explored the role of triamcinolone in reducing adhesions. Our results show decreased adhesions when compared to controls, as well as when compared to published adhesion rates, which range from 40–88% [14,15,62,63,64]. Rates of adhesions in the control group, 71%, were representative of these published rates. Compared to controls, adhesions that were encountered in the study group were fewer in number and density, suggesting that the intervention limited the overall development of adhesions. Removal of posterior fibroids tended to cause higher quantity, density, and more vascular adhesions [65], which were largely omentum or bowel to serosa. This was consistent with observations from other surgeons [14]. However, although triamcinolone instilled intraperitoneally reduces the inflammatory response, it may cause immunosuppression and delayed wound healing [66].

As a result of the decreased rate and extent of adhesions, the study group experienced significantly lower rates of surgical complications during subsequent surgeries when compared to untreated controls and published complication rates [67,68]. Although the incidence of myomas varies by race [69], rates of postoperative adhesion formation tend to be independent of race [70], consistent with our results.

Surgeons have been trying to minimize adhesions through meticulous surgical techniques, minimizing blood loss, shortening surgical time and preoperative heparin, administering anti-inflammatory steroids and antioxidant substances, and using mechanical adhesion barriers [6,44,71,72,73,74,75,76]. Our observations that adhesions were proportionally higher in cases with blood loss >500 mL, operative time >200 min, or >7 uterine incisions reiterate the importance of these factors in reducing fibrosis. Since both hemostasis and separation of tissues seem to be protective from adhesion formation, the technique described [39] involves a combination of these approaches. Heparinized saline irrigation prevents desiccation and blood clots from forming on serosal surfaces, thus limiting adhesion-forming sites. Dextran allows for the separation of tissues that float in a hydroperitoneum and has been shown to decrease adhesion formation [38,77,78].

Our results indicate that EBL and surgical time were proportional to the size, number, and weight of the fibroids removed and the number of adhesions. Factors explaining these findings include a longer time to enter the abdominal cavity when fibroids are large, especially through a Pfannenstiel incision. As each myoma typically requires a separate uterine incision, bleeding and the overall time required to remove the fibroid and sew the defect increases and the area of serosal injury is larger. As the weight of fibroids increases, the defects in the uterus are larger, tend to bleed more, and require more time to repair. Thus, as regions where there is bleeding or foreign materials (such as sutures) increase, the likelihood of adhesion formation increases. Posterior and cervical fibroids are in locations that make myomectomy technically more challenging, and they require more time than anterior or fundal fibroids. Possible explanations for the proportionally larger number of adhesions include the proximity to bowel posteriorly and perhaps the pooling of serosanguinous fluids in the posterior cul-de-sac as the patient lies in recovery.

While our results suggest that triamcinolone may help reduce peritoneal adhesions, it is unclear if this treatment is responsible for changes in the quality of life many years after the initial surgery as patients in the study group reported more pain and bleeding than controls. While further investigation is warranted, this is beyond the scope of this adhesion study.

The strengths of this study include that the cases were performed at a single site using a validated scoring system, which limited inter-surgeon variations in surgical techniques. Weaknesses include that the initial surgery was performed by multiple surgeons. While this paper suggests that the combination of dextran and triamcinolone reduced adhesion formation, the effect of dextran alone compared to triamcinolone alone was not studied in-vivo. Additionally, although the number of fibroids removed was extracted from pathology reports, the number of discrete uterine incisions was not recorded. However, with the limited number of myomectomies performed at this uterine size, the shift in performing minimally invasive myomectomies, and the impracticability of having patients undergo a second-look surgery shortly after the initial procedure, a randomized controlled study may be impractical. Finally, although the sample size calculation required 58 patients to demonstrate a 40% reduction in adhesions, only 52 patients could be analyzed after exclusions for the analysis of the primary outcome variable of the presence of adhesions at the second operation. Nevertheless, we were able to demonstrate a statistically significant decrease in the proportion of patients who had adhesions in the group receiving triamcinolone.

In summary, triamcinolone instilled intraperitoneally during myomectomy significantly reduced the incidence of post-surgical adhesion formation.

### 4.2. Mechanism of Steroids in Adhesion Formation

When the peritoneum is injured, an inflammatory process [79] results in the release of inflammatory cytokines such as profibrotic TGF-β1 [80,81]. This, in turn, helps convert fibroblasts to an adhesion phenotype [82,83]. Studies have shown that the production of ROS [53] from ischemia/hypoxia and/or reperfusion/hyperoxia promotes a cascade of events causing adhesion formation in the injured peritoneum [43,52,84]. If the excessive generation of ROS is decreased through scavengers, adhesion formation is reduced [85]. Oxidative stress, in turn, initiates a HIF-1α-mediated cascade [86,87,88] and causes the conversion of fibroblasts to an adhesion phenotype via a TGF-β—SMAD2/3 pathway [2,43,45,52,54]. Contrary to other investigators, we show that the HIF-1α pathway did not activate the secretion of TGF-β1 in fibroblasts at 1% hypoxia and that HIF-1α was not present at 2% hypoxia where TGF-β1 was upregulated (Figure 2C). This difference may be due multiple factors, including the use of different cell types, hypoxia–reoxygenation, incubation duration, and cell culture media. In our experiments, 2% hypoxia produced a ~35% increase in levels of TGF-β1 and a ~10% increase in ROS. Although it is possible that ROS can act through a signal amplification mechanism, the possibility that other pathways between hypoxia and TGF-β1 exist have not been excluded. Unlike in other tissues, such as brain, our experiments showed that hypoxia generated more ROS than when reoxygenation took place.

Triamcinolone prevented the increase in ROS and the subsequent release in TGF-β1 from 2% hypoxia. We propose that triamcinolone prevents an increase in ROS levels by significantly upregulating mitochondrial function, even in the presence of paraquat (Figure 4C). Paraquat is a redox cycler that may lead to lipid peroxidation in the mitochondrial membrane, thereby impairing the electron transport chain [89,90]. The observation that triamcinolone can upregulate mitochondrial function is consistent with previous reports demonstrating that steroids interact with the inner mitochondrial membrane and increase mitochondrial respiration [91]. We were able to eliminate that triamcinolone itself could scavenge ROS (Figure 4A) or that it was able to upregulate a ROS scavenger (SOD2) or downregulate cytoplasmic ROS generation by NOX2 (Figure 4B).

These results show that the likely mechanism through which intraperitoneal steroid administration works is through an increase in mitochondrial function and a subsequent decrease in ROS formation and an abatement in the irreversible transformation of peritoneal fibroblasts to the adhesion phenotype. We acknowledge the lower replicates for some experiments (reoxygenation) and the use of paraquat as a method to induce ROS. The reoxygenation conditions were used as a proof-of-concept to confirm that the higher TGF-β1 was induced only due to 2% O_2_. Future work will require a focus on determining the precise oxygen tension in the human peritoneum under normal as well as postoperative conditions as, currently, this data remains unknown and has been extrapolated from animal models. Detailed experiments at specific oxygen tensions will precisely characterize the role of triamcinolone under experimental hypoxia to mimic pathophysiology. Multiple mechanisms regulating the electron transport chain may be responsible for triamcinolone’s effects in the mitochondria. Further work will also confirm the source of ROS, seen at 2%, using compartment-specific ROS probes. This will be done as a part of a future study to determine the intramitochondrial factors responsible for this mechanism, where mitochondrial dysfunction leads to an increase in TGF-β1 release and adhesion formation.

In this investigation, we identified differences in TGF-β1 between 1% and 2% hypoxia in vitro. One key differentiating feature between 1% and 2% oxygen is that the former is described as “hypoxia” and the latter as “moderate hypoxia” [92]. The HIF family of proteins has been characterized as the regulators of the hypoxic response in cells. However, one striking feature observed with respect to the function of HIF1α as a transcription factor is that the DNA binding activity of the protein was shown to be maximal in cells maintained between 0.5% and 1% oxygen. The binding activity was negligible at both anoxia (0% oxygen) and 2% moderate hypoxia [93]. In addition, we have previously shown two other transcriptional regulators, MNRR1 [40,94] and CHCHD10 [41], to be maximally activated at 4% and 8% oxygen tension, respectively. Taken together, these results indicate that oxygen responsiveness in vitro is fine-tuned, and there could be specific regulators at different oxygen tensions that function in transcriptional regulation towards the maintenance of homeostasis. Our results indicate that TGF-β1 levels in human fibroblasts are induced at 2% hypoxia. The driver of this response, however, needs to be identified.

## 5. Conclusions

**Figure 5:**
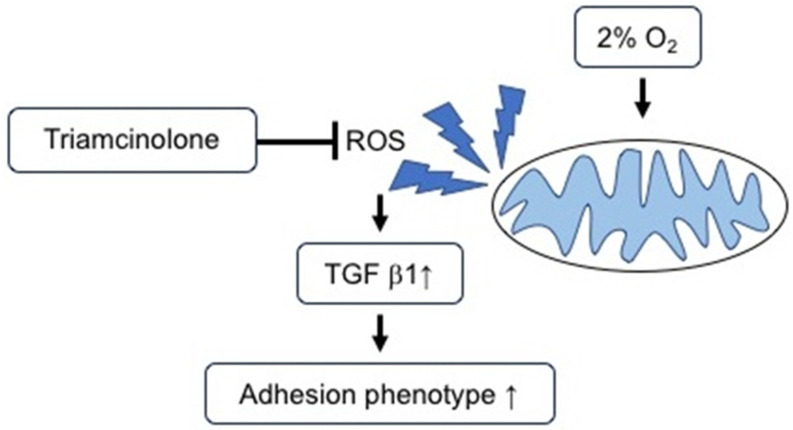
Triamcinolone prevents the increase in ROS and the subsequent release of TGF-β1 at 2% hypoxia, found in the peritoneum.

Fibroid uterus is a common tumor in women of childbearing age, and a common treatment is myomectomy. Minimizing adhesions in patients undergoing abdominal surgery is clinically important. Our data suggest that triamcinolone alters mitochondrial function to prevent the formation of ROS triggered by 2% hypoxia [43,51] and, thereby, abates the transformation of fibroblasts to an adhesion phenotype, as assessed by TGF-β1 secretion (Figure 5). Our cell culture model suggests that the HIF-1α pathway is not the primary means by which fibroblasts convert to the adhesion phenotype. Clinically, therapeutics capable of modulating mitochondrial function in a manner similar to triamcinolone may have a future role in preventing postoperative adhesion formation and improving patient outcomes. Further work is required in this area prior to initiating changes to current clinical practices.

## Figures and Tables

**Table 1:** Adhesion scoring system.

Type of Adhesion	Extent of Total Area
0- None	0- None
1- Filmy, avascular	1- <25%
2- Vascular and/or dense	2- 26–50%
3- Cohesive	3- 51–75%
	4- >76%

**Table 2:** Comparable patient demographics of the study and control populations. Using the intervention significantly reduced the adhesion number, severity, and extent of adhesions. SEM: standard error of the mean.

	Study Population	Controls	*p*
	Mean (SEM)	Mean (SEM)	
**Reoperation (Second Look)**			
Number of patients	31	21	
Age	45.3 (0.8)	44.0 (1.2)	0.321
Race	African American (19), White (8), Hispanic (3), other (1)	African American (9), White (7), Hispanic (3), other (2)	
Mean time to reoperation (years)	7.7 (0.5)	8.1 (0.5)	0.591
Mean uterine size (weeks)	12.0 (0.3)	12.4 (0.5)	0.710
**Original Surgery**			
Age (years)	37.7 (0.9)	36.0 (1.0)	0.257
Avg. original Uterine Size (weeks)	22.1 (0.8)	20.8 (0.8)	0.318
Avg. fibroids removed/patient (#)	7.5 (0.7)	6.8 (0.7)	0.522
Avg. aggregate weight (g)	525 (41.4)	465 (43.9)	0.333
Avg. surgical time (min)	205 (10.5)	237 (18.5)	0.120
Avg. EBL (mL)	624 (90.8)	605 (92.2)	0.885
Total fibroids removed (#)	231		
Fibroid distribution	37% anterior, 20% posterior, 30% fundal, 13% cervical		
**Adhesions**			
Number of patients with adhesions	10 (32%)	15 (71%)	0.006 *
Avg. number adhesions/patient	0.71 (0.2)	2.09 (0.5)	0.006 **
Avg. severity of adhesions/patient	0.54 (0.2)	1.38 (0.2)	0.005 ***
Avg. extent of adhesions/patient	0.45 (0.1)	1.28 (0.2)	0.003 ****
Location	40% multiple sites, 60% posterior	67% multiple sites, 20% posterior	
# Surgical Complications at second surgery	1 (bladder injury)	3 (2 bladder, 1 bowel injury)	

* Power of test for proportion difference: 0.809. ** Power of test for proportion difference: 0.672. *** Power of test for proportion difference: 0.809. **** Power of test for proportion difference: 0.814. (#: denotes number).

**Table 3:** Effectiveness of the intervention by stratified group. Stratifying the study group, the intervention was particularly effective at preventing adhesion formation when estimated blood loss, operative time, number of fibroids removed, and uterine size were smaller. Mean (standard error of the mean) and significance are reported.

**Estimated Blood Loss**	**<500 mL**	**>500 mL**	***p***
N size	15	16	
Uterine size (weeks)	18.5 (0.83)	25.3 (0.72)	10^−6^
Fibroids removed	4.6 (0.66)	10.2 (0.82)	10^−^^5^
Aggregate weight (g)	347 (39)	693 (37.9)	10^−^^7^
Posterior fibroid	0.6 (0.21)	2.37 (0.35)	0.002
Cervical fibroid	0.13 (0.09)	1.81 (0.36)	0.001
Operative time (min)	164 (11.5)	244 (10.35)	10^−5^
Adhesion number	0 (0)	1.37 (0.36)	0.001
**Operative Time**	**<200 min**	**>200 min**	***p***
N size	13	18	
Uterine size (weeks)	17.8 (0.799)	25.11 (0.67)	10^−7^
Fibroids removed	3.8 (0.57)	10.1 (0.69)	10^−7^
Aggregate weight (g)	314.9 (37.6)	677.0 (35.1)	10^−7^
Posterior fibroid	0.53 (0.26)	2.23 (0.31)	0.005
Cervical fibroid	0.15 (0.1)	1.61 (0.35)	0.007
Estimated blood loss	219.2 (46.2)	916.67 (109.44)	10^−^^6^
Adhesion number	0 (0)	1.22 (.34)	0.005
**Fibroids Removed**	**≤7**	**>8**	***p***
N size	16	15	
Uterine size (weeks)	19.1 (0.96)	25.2 (0.69)	10^−5^
Aggregate weight (g)	376 (48.3)	685 (37.1)	10^−^^6^
Operative time (min)	162 (9.7)	251.5 (8.4)	10^−^^7^
Estimated blood loss	303.1 (59.6)	966.7 (127.4)	0.001
Adhesion number	0 (0)	1.46 (0.37)	0.001
**Uterine Size (Weeks)**	**<22**	**≥22**	***p***
N size	12	19	
Fibroids removed	4.1 (0.66)	9.63 (0.78)	10^−6^
Aggregate weight (g)	292.2 (34.4)	673 (32.8)	10^−^^8^
Operative time (min)	148.4 (8.5)	241.5 (9.4)	10^−^^8^
Estimated blood loss	225 (60.1)	876 (109.1)	10^−^^5^
Adhesion number	0 (0)	1.16 (0.33)	0.009

## Data Availability

The datasets that support the findings of this study are available from the corresponding author (M.-A.R.) upon reasonable written request.

## Supplementary Materials

The following supporting information can be downloaded at: https://www.mdpi.com/article/10.3390/jcm11020301/s1, Figure S1: In the study group, adhesion severity found at second-look is related to surgical blood loss, surgical time and number of fibroids removed. Table S1: Severity of Adhesions as Determined During Second Surgery.
