# Supplementary material for: Intraperitoneal Triamcinolone Reduces Postoperative Adhesions, Possibly through Alteration of Mitochondrial Function
Source: J Clin Med. 2022 Jan 7;11(2):301. doi: 10.3390/jcm11020301 (PMC8779954; doi:10.3390/jcm11020301)
Supplement: Supplementary file 1 [file jcm-11-00301-s001.zip › jcm-1519186-supplementary.pdf]

Table S1. Severity of Adhesions as Determined During Second Surgery.

	**r**	**p**
Uterine size	0.54	0.002
Fibroids removed	0.79	10^{-8}
Aggregate weight (g)	0.60	10^{-4}
Posterior fibroid	0.78	10^{-7}
Cervical fibroid	0.79	10^{-8}
Surgical time (m)	0.69	10^{-5}
EBL	0.77	10^{-7}
Adhesion Number	0.92	10^{-13}

Pearson correlation n = 31.

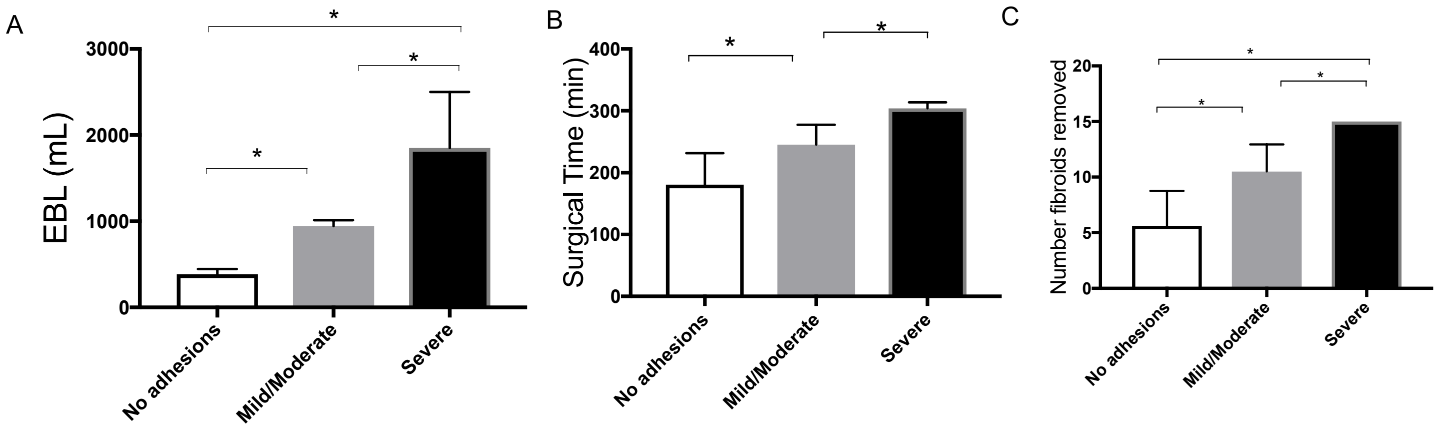

Figure S1. In the study group, adhesion severity found at second-look is related to surgical blood loss (**A**) surgical time (**B**) and (**C**) number of fibroids removed at initial surgery. The three groups included, no adhesions (adhesion score of 0), $n = 21$; mild/moderate (scores of 1 and 2), $n = 8$; and severe adhesions (score ≥ 3), $n = 2$. * denotes significance with $p < 0.05$.
